# Subjective assessment of sensory function and oral function decline in older adults

**DOI:** 10.1371/journal.pone.0326788

**Published:** 2025-06-27

**Authors:** Tetsuo Ichikawa, Tomoya Koda, Mio Kitamura, Takahiro Kishimoto, Takashi Matsuda, Takaharu Goto, Masayuki Domichi, Akiko Suganuma, Shinji Fujiwara, Yasuhiko Shirayama, Kazuhiko Kotani, Naoki Sakane

**Affiliations:** 1 Department of Prosthodontics & Oral Rehabilitation, Tokushima University Graduate School of Biomedical Sciences, Tokushima, Japan; 2 Division of Community and Family Medicine, Jichi Medical University, Tochigi, Japan; 3 Kamikatsu Town Clinic, Tokushima, Japan; 4 Department of Community Medical and Welfare, Tokushima University Graduate School of Biomedical Sciences, Tokushima, Japan; 5 Division of Preventive Medicine, Clinical Research Institute, National Hospital Organization Kyoto Medical Center, Kyoto, Japan; 6 Mima Municipal Koyadaira Clinic, Tokushima, Japan; Waseda University: Waseda Daigaku, JAPAN

## Abstract

Sensory decline in older adults significantly affects quality of life and contributes to cognitive decline, depression, falls, and injuries. Although several studies exist in this area, most were focused on individual senses, with few being conducted on comprehensive assessments of all five senses. The aim of this study was to investigate the relationship between subjective sensory assessment and oral function, to developing health strategies. This study was conducted as part of the Mima-SONGS Study for examining relationships between oral, cognitive, and physical functions, social factors, nutrition, and health, in older adults living in a mountainous region of Japan. The cohort included 62 participants (40 women and 22 men; mean age: 80.8 yrs.) as of December 2023. Participants completed a questionnaire assessing sensory perception and eating enjoyment rated on a four-point scale. Oral health was evaluated based on the conditions of remaining teeth, tongue coating, oral dryness, occlusal force, oral diadochokinesis, and repetitive salivary swallow test. Sensory assessments indicated minimal overall issues, with auditory problems scoring the highest and taste/tactile issues scoring the lowest. Males scored higher in hearing and maximum occlusal force. Eating enjoyment was generally high and negatively correlated with olfactory and taste problems. Subjective sensory issues were less strongly associated with oral function and age. Most older adults were not subjectively aware of sensory problems, especially olfaction, taste, and tactile problems. Subjective sensory problems showed a moderate but meaningful association with oral health conditions and age. The findings might be valuable data developing future support measures.

## Introduction

Decline in sensory functions, including vision and hearing, in older adults has garnered increasing attention in recent years owing to its significant impact on quality of life and health. The World Health Organization Guideline recommends regular visual and auditory examinations, as well as appropriate care, to help older individuals maintain their physical and mental health and independence [1]. Declining sensory function has been shown to cause difficulties in daily activities, exacerbate feelings of isolation, and increase the risk of developing depressive symptoms [2–4]. Notably, visual and hearing impairments are linked to reduced cognitive function and increased risk of dementia [5].

Furthermore, olfactory and gustatory disturbances, frequently reported as sequelae of severe acute respiratory syndrome infection, have been shown to have a substantial impact on quality of life, particularly in older adults [6,7]. Decline in sensory functions can lead to reduction in meal enjoyment and negatively affect dietary quality. Additionally, tactile senses such as touch and warmth play a crucial role in defensive responses and self-awareness. Their decline has been associated with elevated risk of physical injuries, including falls and burns [8,9].

Despite the proliferation of research in this area, most studies on sensory function and health in older adults have been focused on individual senses, with few being conducted on comprehensive assessments of all five senses (vision, hearing, olfaction, taste, and tactile). In addition, although a discrepancy between objective sensory impairment and subjective symptoms has been reported in previous studies, the relationship between subjective assessments of sensory impairment and oral function involving many senses has not been examined in detail. The objective of this study was to investigate the relationship between sensory and oral functions by subjectively assessing all five sensory functions, as part of a cohort study conducted in a mountainous region. The aim was to provide valuable insights into the impact of sensory decline on the health and quality of life of older adults, contributing to the development of potential strategies for prevention and intervention.

## Materials and methods

### Research field and participants

This study was conducted as part of the “Prospective Study on the Effects of Shopping, Oral Function, Nutrition, and Genetics on Sarcopenia, Frailty, and Healthy Life Expectancy in Mima City” (Mima-SONGS Study; Principal Investigator: Shinji Fujiwara), a cohort study of older adults living in mountainous areas. The aim of the study was to examine the relationships among oral function, cognitive function, physical function, social factors (e.g., participation in community activities), nutritional intake, and health. This study was initiated in 2018 with the goal of developing a comprehensive approach to extend the healthy life expectancy of older adults. The study area, Koyadaira in Mima City, is located in western Tokushima Prefecture, where approximately 95% of the terrain is mountainous. As of 2018, Koyadaira had a population of 645, with an aging rate of more than 60%.

Sixty-two outpatients aged 65 years or older (mean age: 80.9 ± 6.03 years) who were available for study participation in December 2023 were included. The inclusion criterion for participants was the ability to communicate verbally with the attending physician in a routine outpatient clinic setting. Written informed consent was obtained from all participants prior to the study. The study was approved by the Kyoto Medical Center Ethics Committee (approval number: 17-032) and conducted in accordance with the ethical guidelines outlined in the Declaration of Helsinki.

### Questionnaire for subjective assessment of sensory perception and eating enjoyment

The questionnaire consisted of 10 items evaluating participants’ current subjective perceptions of vision, hearing, olfaction, taste, and tactile capacities, as well as a retrospective assessment comparing current sensory abilities with those in the past (Table 1). The examiner showed each question to the participants, read the questions aloud, and confirmed their answers. Responses were rated on a four-point scale: 1 = Not applicable, 2 = Tends to be the case, 3 = Sometimes the case, and 4 = Fully applicable. Given the inherent complexities in the subjective evaluation of tactile sensations, and recognizing the heterogeneity in cognitive function and comprehension among the older adult participants in this study, the retrospective assessment employed simplified and easily memorable expressions—such as the common experience of feeling “hot or cold.” In the current assessment, more direct and readily identifiable sensory descriptors, including “discomfort” or “numbness,” were introduced to better capture immediate tactile abnormalities. This modification aimed to enhance the accuracy of participant responses by accounting for potential variations in understanding. Eating enjoyment using all five senses was also assessed on the same four-point scale.

**Table 1:** Questionnaire for subjective assessment of sensory perception and eating enjoyment.

Assessments	Questions
Retrospective assessment comparing current sensory perceptions to those in the past	I used to be able to see, but now I can’t
	I used to be able to hear, but now I can’t
	I used to be able to smell, but now I can’t
	I used to be able to taste well, but now I can’t
	I used to feel hot/cold well, but now I can’t
Current subjective assessment	Currently I currently have difficulty seeing.
	Currently I have difficulty hearing.
	Currently I have difficulty in picking up smells or have strange smells.
	Currently I have difficulty tasting or have strange tastes.
	Currently I feel discomfort in mouth or feel numbness.
Eating enjoyment	I enjoy eating everyday.

Evaluation: 1 = Not applicable, 2 = Tends to be the case, 3 = Sometimes the case, and 4 = Fully applicable.

### Measurements of oral conditions

Oral conditions were determined by examining the number of remaining teeth, number of occluded teeth, tongue coating index (TCI) [10], oral dryness, and salivary characteristics by one examiner. The number of remaining teeth including residual roots, was counted. The number of occluded teeth was counted as one if the upper and lower teeth were in occlusal contact. Oral dryness was evaluated on a 3-point scale: 0 (no problem), 1 (dryness), and 2 (obvious dryness). Salivary characteristics were evaluated at three levels: 0 = no problem, 1 = viscous, and 2 = obviously viscous.

Maximum occlusal force was measured using the Dental Prescale II (G.C., Tokyo, Japan) [10]. Participants were instructed to sit with their heads unsupported by a headrest, ensuring that the Frankfurt plane was parallel to the floor, and to bite the measuring device with maximum force for 3 seconds. The recorded data were analyzed using a bite force analyzer (G.C., Tokyo, Japan). Oral diadochokinesis was assessed by instructing participants to repeat “pa,” “ta,” and “ka” as quickly as possible for 5 seconds.^10^ The repetitions were converted to a per-second rate using dedicated software. In the repetitive salivary swallow test (RSST), participants were asked to swallow saliva as many times as possible within 30 seconds [11]. The examiner palpated the hyoid bone with the second finger and the thyroid cartilage with the third finger. Only movements in which the thyroid cartilage overcame both fingers were recorded.

Each measurement was conducted once per participant by the same examiner to ensure consistency. Thirteen patients (21.0%) had missing molars in either the maxilla or mandible. For participants who wore dentures, all measurements were recorded with their dentures fully placed in the oral cavity.

### Statistical analysis

All statistical analyses were performed using SPSS® version 29.0 (IBM, Chicago, IL, USA). Sex differences were assessed using the Mann–Whitney U test, whereas the Wilcoxon signed-rank test was used to compare questionnaire items. Correlations between age and each factor were evaluated using Spearman’s rank correlation coefficient. The significance level for all the tests was set at 5%.

Associations between oral function and the five sensory functions were analyzed using partial correlation coefficients with sex as a control variable. Binomial logistic regression was used to identify oral function factors associated with each sensory function. For this analysis, participants were divided into two groups: an upper group, which included participants with fewer problems in each sense (scores < 4), and a lower group, which included participants with more problems (scores ≥ 4). The goodness-of-fit for the logistic regression models was assessed using the Hosmer–Lemeshow test, with a threshold of 5% or greater.

## Results

Forty females (mean age 81.4 ± 6.14 years) and 22 males (mean age 80.0 ± 5.85 years) were included in the analysis.

The means and standard deviations of the scores for the sensory questions are presented in Table 2. Among these, only the score for hearing assessments fell within the 2-point range (2.3 ± 1.28 for females, 3.1 ± 1.17 for males, and 2.6 ± 1.30 overall); all other scores were within the 1-point range, with particularly low scores for taste and tactile sensations, indicating that respondents experienced minimal sensory issues. For vision, hearing, and olfaction, the scores for current inconvenience were significantly lower than those for retrospective assessments, suggesting that sensory assessments may be influenced by habituation. Comparisons between male and female participants revealed that males tended to have higher scores than did females, particularly for hearing, for which their score was significantly higher. Additionally, significant correlations were found between taste and olfaction (ρ = 0.306; p < 0.05) and between tactile and vision (ρ = 0.469; p < 0.01).

**Table 2:** Results of subjective assessment.

Sense	Female		Male	
	Retrospective assessment	Current assessment	Retrospective assessment	Current assessment
Vision^†^	1.8 ± 1.17	1.6 ± 1.04	2.1 ± 1.38	1.9 ± 1.38
Hearing^††^	2.3 ± 1.28	1.7 ± 0.97	3.1 ± 1.17*	2.6 ± 1.34*
Olfaction^†^	1.4 ± 0.98	1.1 ± 0.52	1.6 ± 1.10	1.5 ± 1.06
Taste	1.3 ± 0.74	1.2 ± 0.72	1.4 ± 0.79	1.2 ± 0.75
Tactile	1.0 ± 0.16	1.1 ± 0.22	1.4 ± 1.05	1.2 ± 0.69

The values represent means and standard deviations.

* p < 0.05: Mann–Whitney U test. Comparison between sexes.

† p < 0.05, ^††^ p < 0.01: Wilcoxon signed-rank test. Comparison between two assessments.

The means and standard deviations of the basic attributes of the participants and the measurements of oral function are shown in Table 3. Males had significantly higher scores than did females for maximum occlusal force and RSST. No sex differences were observed for other measurement items. The measurement values themselves were not considered to be a significant problematic older group, considering the age of the participants. Subjective sensory assessment was not related to age; however, factors related to oral function, except for TCI and RSST, were significantly negatively correlated with age.

**Table 3:** Basic attributes and measurements on oral function factors.

	Female	Male
Sample No.	40	22
Age (y)	81.4 ± 6.14	80.0 ± 5.85
Remaining teeth No.	10.5 ± 10.93	11.3 ± 11.52
Occluded teeth unit	4.0 ± 5.53	4.2 ± 5.70
TCI	38.3 ± 27.50	51.0 ± 28.00
Oral dryness	0.2 ± 0.41	0.2 ± 0.40
Saliva condition	0.1 ± 0.22	0.1 ± 0.21
Maximum occlusal force (N)	465.9 ± 343.28	722.1 ± 489.18*
ODK/pa/	5.5 ± 1.10	5.1 ± 1.33
ODK/ta/	5.5 ± 1.19	5.2 ± 1.20
ODK/ka/	5.3 ± 1.20	4.8 ± 1.24
RSST	2.6 ± 1.03	4.5 ± 1.26**
Eating enjoyment	3.4 ± 0.95	3.2 ± 0.87

The values represent means and standard deviations.

TCI: Tongue coat index, ODK: Oral diadokokinesis, RSST: Repeated saliva swallowing test.

*p < 0.05, **p < 0.01: Mann–Whitney U test. Comparison between sexes.

The mean and standard deviation of eating enjoyment scores were 3.3 ± 0.92, indicating high values. Eating enjoyment showed significant negative correlations with olfactory (ρ = −0.292; p < 0.05) and taste (ρ = −0.314; p < 0.05) assessments but no significant correlation with oral factors.

Binomial logistic analysis of the factors affecting each sensory problem, with sex, age, and oral function as independent variables, yielded the results shown in Table 4. Number of remaining teeth was determined to be relevant in hearing and taste assessments, and oral dryness was associated with olfaction.

**Table 4:** Relationship of subjective assessments of five senses with oral function factors.

Sense	Number of applicable examinees		Extracted factors (odds ratios)	Hosmer & Lemeshow test
	Upper group	Lower group		
Vision	44	18		0.980
Hearing	30	32	Remaining teeth (1.289) Gender (14.647)	0.812
Olfaction	54	8	Oral dryness (14.145)	0.670
Taste	56	6	Remaining teeth (1.370)	<0.01
Tactile	58	4		0.000

Upper group included participants with fewer problems in each sense (scores < 4).

Lower group included participants with more problems (scores ≥ 4).

Extracted factors were identified using binominal logistic regression. The dependent variable was each sense, and the independent variables were oral function factors in Table 3.

## Discussion

Decline in sensory functions and impairments in sensory organs, including teeth, in older adults not only directly reduces the quality of life but is also linked to various diseases. For example, hearing loss is the highest-ranking risk factor for dementia [5]. Furthermore, reduced social connectedness has been shown to be associated with increased mortality and functional disability [12]. It will be reasonable to infer that a decline in sensory function and sensory organ impairment contributes to reduced social connectedness.

In terms of aging sensory functions, particularly hearing, the prevalence of hearing loss has been reported to be 71.4% in males and 67.3% in females aged 75–79 years, and 84.3% in males and 73.3% in females aged 80 years and older [13]. Decline in high-frequency hearing is particularly pronounced with aging [14]. However, only 34.4% of older adults aged 75 years or older are aware of their hearing loss [15], highlighting the low level of awareness of this condition. With regard to vision, 47.3% of male and 42.6% of female aged 70 years and older reported having visual impairments [16]. Visual impairment is considered a factor that significantly reduces quality of life and independence, and it has been pointed out that it may limit daily activities (reading, driving, housework, etc.) and increase the risks of social isolation and depression. In addition, recent studies have suggested that reduced vision is associated with reduced cognitive function, possibly due to reduced sensory stimulation of the brain caused by visual impairment and the effects of psychological and social factors caused by loss of vision [1,3,17,18]. Regarding olfaction, the Beaver Dam Offspring Study revealed that 13.9% of adults aged 65 years and older were aware of their olfactory impairment, and the prevalence increased with age [19]. Among individuals aged 80–97 years, 62.5% had olfactory impairment [20]. Olfactory dysfunction has been identified as an early indicator of neurodegenerative diseases, including Alzheimer’s and Parkinson’s diseases. This condition is thought to arise from degenerative changes in the olfactory nerves, abnormal neurotransmission in the brain, or other physiological factors [21,22]. Taste sense also declines with age, and in particular, a decrease in saltiness sensitivity has been noted. These changes are thought to be due not only to a decrease in the number of taste buds, which are sensors of taste perception, but also to changes in saliva properties, dryness of the oral cavity, and other factors [22–24]. The effects of aging on tactile sense has been a subject of significant interest. Various factors, such as alterations in saliva composition, oral dryness, and changes in the mechanoreceptors distributed on the tongue surface, have been implicated in age-related changes in tongue tactile sensation [25,26].

In this study, although many participants were likely to have sensory impairments owing to their age, few older adults were aware of these impairments, particularly those related to oral sensory function. In contrast, they were relatively more aware of auditory and visual issues. A comparison of retrospective subjective assessments of sensory function with current assessments showed that the latter were significantly lower. While oral motor functions, such as lip and tongue movements and occlusal force, showed significant correlations with age, which is consistent with the results of previous studies [27,28], no correlation was found between subjective sensory assessments and age. The subjective evaluation of difficulty in chewing is related to both age and oral factors in older adults [29], and a decline in oral function may lead to a decrease in eating enjoyment due to difficulty in chewing. However, in this study, no direct impact was observed. Furthermore, although subjective assessment of olfaction was shown to be associated with oral dryness, no significant associations were found between subjective assessments of sensory function and oral factors. These findings suggest that the older adults are accustomed to declining sensory function and accept sensory decline like “Gero-transcendence [30,31].” This “acceptance” or “adaptation” mechanism may be part of the psychological adaptability of older adults. However, when asked whether they enjoy eating every day, subjective problems with taste and smell were shown to have an impact, confirming the influence of sensory function on quality of life with regard to food enjoyment. Regarding the relationship between subjective assessments of sensory functions, the correlation between olfactory and taste problems may be because these senses cannot be clearly distinguished and may be considered as problems related to food intake. The reason for the significant correlation between vision and tactile senses is unclear, but it may be related to a kind of “cross-modal plasticity,” in which the lack of visual information increases anxiety about what one eats [32]. The observation that our study participants demonstrated a potential effect of geriatric transcendence on sensory decline may be attributable to the characteristics of the mountainous region in which the study was conducted. In this area, access to essential aspects of daily life—such as medical care, nursing services, and shopping—is limited. As a result, a culture of mutual support and community interdependence may continue to thrive. The positive subjective sensory assessments observed in our study suggest that these close-knit community relationships could have a beneficial impact on both sensory function and the overall well-being of older adults.

Additionally, the limited access to dental care in this region warrants attention. The scarcity of professional dental services may lead to fewer opportunities for older adults to receive objective evaluations and appropriate management of their declining oral function. This lack of professional input could, in turn, contribute to reduced awareness of their actual oral health status, potentially explaining the discrepancy observed between subjective sensory assessments and objective oral function in our findings.

This study has several limitations. It was conducted in a single region with a relatively small sample size, and therefore caution is warranted when generalizing the results. A total of 62 participants aged 65 years or older were included. A post hoc power analysis was performed using G*Power software (version 3.1.9.7, Heinrich-Heine-University, Düsseldorf, Germany). With an alpha level (α) of 0.05 and an effect size of 0.52 (calculated from the observed means and standard deviations), the statistical power of the study was 0.98, indicating that the sample size was adequate to detect the observed effect. However, this study utilized binomial logistic regression analysis. Previous research has suggested that when the sample size of the smaller category of the dependent variable is less than ten times the number of explanatory variables, issues such as estimation bias, reduced precision, and poor model fit may arise [33]. In our analysis, the sample sizes of the smaller outcome categories ranged from 4 to 32, suggesting a potential for such limitations. Therefore, we acknowledge the risk of bias and reduced precision in our findings due to these small subgroup sizes. Nonetheless, the results of this study may still be meaningful. The study population comprised nearly all older adults who could be examined at the only clinic in a mountainous region with a population aging rate exceeding 60%. This unique regional context may offer valuable insights into aging and oral health in underserved areas. Future studies should aim to include a broader range of geographical regions with similar demographic characteristics to validate and expand upon these findings.

Furthermore, this study relied solely on subjective sensory evaluation, rather than objective assessments. This methodological choice stemmed from two primary considerations within the context of our older study population and the Mima-SONGS project’s aims. First, the practical challenges of implementing comprehensive objective sensory testing across all five senses in an epidemiological study involving older adults in a rural mountainous region were substantial. Objective assessments, particularly for tactile, thermal, taste, and olfactory functions, are often time-intensive and require specialized equipment and trained personnel, posing logistical difficulties for a cohort study with potential access limitations and participant burden. Conversely, basic oral function tests were more readily integrated into our existing protocol due to their shorter administration times. Second, our focus on subjective evaluation was also informed by existing literature, as noted in the Introduction, which frequently reports discrepancies between objective sensory impairment and subjective awareness in older adults. The relationship between subjective sensory impairment and a comprehensive evaluation of all five senses remains understudied. Consequently, we addressed this gap by concentrating on the subjective evaluation of the five senses in this study. However, it is important to acknowledge potential communication issues that might have influenced the subjective evaluations, given the age of our participants and the lack of formal validation for the questionnaire.

It was conducted in an isolated mountainous area, the study revealed the state of awareness regarding sensory function among older adults living in a highly independent mountainous area. This study provides valuable data for developing future support measures for older adults. To further build on these findings, follow-up surveys should be conducted in diverse regions and environments to gather additional evidence.

## Conclusions

Most older adults in an isolated mountain community were not subjectively aware of sensory problems, especially olfaction, taste, and tactile impairments. Subjective assessments of sensory functions showed a moderate but meaningful association with oral health conditions and age.
